# Circumpolar Inuit health systems

**DOI:** 10.3402/ijch.v72i0.21402

**Published:** 2013-08-05

**Authors:** Leanna Ellsworth, Annmaree O'Keeffe

**Affiliations:** Inuit Circumpolar Council, Ottawa, ON, Canada

**Keywords:** Inuit health, Inuit health systems, Arctic health, indigenous health

## Abstract

**Background:**

The Inuit are an indigenous people totalling about 160,000 and living in 4 countries across the Arctic – Canada, Greenland, USA (Alaska) and Russia (Chukotka). In essence, they are one people living in 4 countries. Although there have been significant improvements in Inuit health and survival over the past 50 years, stark differences persist between the key health indicators for Inuit and those of the national populations in the United States, Canada and Russia and between Greenland and Denmark. On average, life expectancy in all 4 countries is lower for Inuit. Infant mortality rates are also markedly different with up to 3 times more infant deaths than the broader national average. Underlying these statistical differences are a range of health, social, economic and environmental factors which have affected Inuit health outcomes. Although the health challenges confronting the Inuit are in many cases similar across the Arctic, the responses to these challenges vary in accordance with the types of health systems in place in each of the 4 countries. Each of the 4 countries has a different health care system with varying degrees of accessibility and affordability for Inuit living in urban, rural and remote areas.

**Objective:**

To describe funding and governance arrangements for health services to Inuit in Canada, Greenland, USA (Alaska) and Russia (Chukotka) and to determine if a particular national system leads to better outcomes than any of the other 3 systems.

**Study design:**

Literature review.

**Results:**

It was not possible to draw linkages between the different characteristics of the respective health systems, the corresponding financial investment and the systems’ effectiveness in adequately serving Inuit health needs for several reasons including the very limited and inadequate collection of Inuit-specific health data by Canada, Alaska and Russia; and second, the data that are available do not necessarily provide a feasible point of comparison in terms of methodology and timing of the available data collection.

**Conclusions:**

Despite the variations in the health systems as well as national, political and economic approaches, none is adequately addressing Inuit health needs. All Inuit populations still have significant gaps between their health status and those of broader national populations. Meaningful measurement and evaluation of the effectiveness of the respective health systems is severely hindered by the lack of relevant, Inuit-specific health data. The inadequacy, and in a number of cases absence of relevant data, hinders the design and development of a better and potentially more effective approach to delivering health services to Inuit.

The Inuit^1^ are an indigenous people totalling about 160,000 and living in 4 countries across the Arctic – Canada, Greenland, USA (Alaska) and Russia (Chukotka). Although the health challenges confronting the Inuit are in many cases similar across the Arctic, the responses to these challenges vary in accordance with the types of health systems in place in each of the 4 countries. This article provides an overview of those systems with particular relevance to the Inuit populations in each country. The data and findings draw on a range of literature that has looked at Arctic health and reflects statistics found in these sources as well as databases.

Although there have been significant improvements in Inuit health and survival over the past 50 years (1), stark differences persist between the key health indicators for Inuit and those of the national populations in the United States, Canada and Russia and between Greenland and Denmark (see Tables I and II). On average, life expectancy in all 4 countries is lower for Inuit. Infant mortality rates are also markedly different with up to 3 times more infant deaths than the broader national average. Underlying these statistical differences are a range of health, social, economic and environmental factors which have affected Inuit health outcomes.

**Table I:** Life expectancy for Inuit and national populations

Indicator	USA 2000–2004*	Alaska Natives 2000–2004*	Canada 2001**	Inuit 2001**	Denmark 20008***	Greenland 2008***	Russia 2008****	C'kotka '00–‘04*
Life expectancy at birth	M 74.6	M 68.1	M 77.2	M 64.4	M 76.3	M 66.6	M 61.8	M 53.6
	F 80.0	F 75.4	F 82.2	F 69.8	F 80.7	F 71.6	F 74.1	F 63.7

* Circumpolar Health Indicators: Sources, Data and Maps; T Kue Young; Circumpolar Health Supplements, 2008.

** Inuit Statistical Profile, Inuit Tapiriit Kanatami, 2008.

*** Health Statistics in the Nordic Countries, Nordic Medico-Statistical Committee (Nomesco) 2010.

**** WHO Country data, 2011.

**Table II:** Same-time comparative health indicators for Inuit and national populations

Indicator (2000–2004)	USA	Alaska natives	Canada	Nunavut	Denmark	Greenland	Russia	C'kotka
Life expectancy at birth	M 74.6	M 68.1	M 77.2	M 66.6	M 74.9	M 63.7	M 58.8	M 53.6
	F 80.0	75.4	F 82.2	F 70.0	F 79.6	F 70.0	F 72.1	F 63.7
Infant mortality/1,000 live births	6.9	10.8	5.3	15.3	4.7	12.1	13.3	20.3

Source: Circumpolar Health Indicators: Sources, Data and Maps; T Kue Young; Circumpolar Health Supplements, 2008.

This inequity between Inuit and broader national populations is consistent with the poor health status of indigenous peoples globally. It is increasingly recognized that the differentiation is due to a range of factors – physical, psychological and social and reflecting broader contexts including historical, economic and environmental. It is also a reality that “gaps between indigenous and non-indigenous peoples are not only clear in health status, but also in socio-economic status, education, employment, environmental and social health and most other social determinants of health” (2).

## Materials and methods

A comprehensive and systematic literature search was conducted focusing on the variations between Arctic health systems and in particular those delivering health services to Inuit populations in Canada, Greenland, USA (Alaska) and Russia (Chukotka). Databases managed by the World Health Organization (WHO), World Bank and NOMESCO as well as other databases relevant to Arctic health were also accessed.

The material obtained from the search was then used to undertake a comparison of the differences between the 4 national systems and to observe any noticeable impact of the differences on Inuit health outcomes. Each of the 4 countries has a different framework for national health care and within these systems, there are variations in the way in which health services are delivered to indigenous and in particular Inuit population with varying degrees of accessibility and affordability for Inuit living in urban, rural and remote areas.

Drafts of the review were provided for feedback and comment to members of the Circumpolar Inuit Health steering committee, which has Inuit health expert representation from each of the 4 countries.

## Results and discussion

### Country health systems

Overall, responding to the health needs in each of the 4 countries varies in line with each country's economic and political framework as well as the different health systems.

In Canada, national, government-funded universal health care is administered by territories and provinces with health care funding a mix of largely public and some private. In Alaska, health care is mostly private with personal health insurance an important feature of the funding arrangements. However, within both countries, there are specific government-funded arrangements to support indigenous health.

In Greenland, where the population is predominantly Inuit, there is universal government-funded coverage. The Russian system is working through the challenges of shifting from a government-controlled and funded system to a decentralized insurance-based framework.

Also affecting the effectiveness of the health system response is the isolation of many of the Inuit communities from health and education services, which are more accessible to larger urban-based populations. It is important to note that the relative smallness of the northern populations includes not just Inuit. Remoteness, distance and the smallness of the communities all play their part in shaping the accessibility, availability and quality of the health services for northern populations including Inuit. For example, Canada's northern population is just over 101,000 including approximately 39,000 Inuit. This constitutes less than 0.5% of Canada's total population (3). Alaska's Inuit population represents only 7% of the state's total population or half the total Alaskan native population. In Russia, there are 40 different indigenous peoples in the north totalling about 280,000 or 0.2% of the national population; of these, the 1,750 Inuit represent less than 1% of the total indigenous population (4). Only in Greenland are Inuit the majority representing 87% of the population.

Cost per capita on the other hand appears to reflect the expense of delivering services in remote and sparsely populated regions but not necessarily improved availability and services. For example, Nunavut in Canada has the highest per capita health expenditures in the world at just under 26% of the territory's GDP.

A major on-going challenge for health delivery to Inuit communities is the recruitment of health and allied professionals able and willing to work in the remote and isolated communities. Aligned to this challenge is the on-going difficulty in ensuring that staff who are working in the Arctic have the medical skills and cultural knowledge appropriate for the region.

### Greenland

#### Population

A total of 57,637 (July 2010 estimate) of whom 85% live in urban centres (5); 87% of the population is of Inuit origin.

#### Geographic and political profile

Greenland is the world's largest island with the vast majority of Greenlanders living along the fjords on the central and southwestern coast. Many live in urban centres but Greenland is the least densely populated country in the world with the towns and villages isolated from each other and accessed only by air or sea. A colony of Denmark since 1721, the passing of the Act on Greenland Self Government in the Danish parliament in June 2009 has brought Greenland closer to full independence. Under the Act, Greenland receives an annual subsidy (3,439.9 million DKK in 2009 or approximately US$ 630 million), which represents about 60% of the government's annual revenue. The amount is adjusted annually in accordance with general price and wage increases. The official languages of Greenland are Danish and Greenlandic, which is closely aligned to the Inuit language spoken in Alaska, Canada and Chukotka, Russia. It is important to note, however, that within the health system, Danish is the dominant language spoken because a large proportion of the medical staff are recruited from Denmark.

#### Profile of health system

Fully government-funded, universal health care.

#### Health challenges

The leading health challenges confronting Greenland are high infant mortality rate; high rates of suicide, child abuse, abortion and accidents; high rates of infectious disease, notably tuberculosis, hepatitis B, sexually transmitted diseases, *Helicobacter pylori* and meningitis; increasing rates of diabetes, cardiovascular diseases and cancers; substance abuse; low oral health; and contaminated traditional diet.^2^

#### Responsible authorities for health services

Health services and funding is a shared responsibility between the Greenland Government and the municipalities. The Ministry of Health is responsible for legislation and overall management. The National Board of Health is responsible for supervision of health services and clinical guidelines. Health authorities are responsible for running the clinical services including primary health, specialized services, distribution of pharmaceuticals, nursing care, home nursing services in some districts, home mental health care, preventive services, rehabilitation, and child and school health services (6).

The municipalities are responsible for home nursing services in some health districts, preventive services and nursing homes. They are also principally responsible for services that support health and well-being including social services, basic education, funding for cultural and sporting events and the local environment. Plans are underway to transfer responsibility for the treatment of alcohol and drug abuse and services for the disabled to the municipalities (7). Also, as part of the health reform process, the municipalities were united into 5 health regions from 2011. Dental services are provided free of charge in public dental clinics and there is limited access to private dentists where treatment is paid for by the patient. Greenland's government owns the hospitals and there are no private or specialized hospitals. There is no free choice of hospital in Greenland. Patients are referred by the district hospitals for treatment at the National Hospital and a special committee refers patients for treatment outside Greenland. Medicines are free and dispensed by the health services (8).

Nuuk has a central hospital for specialized treatment but more intensive care or specialized treatment is conducted in Copenhagen. The district medical centres are autonomous units. Depending on the size of the population, the centres will have between 1 and 5 doctors plus nurses, midwives, health care assistants, lab-technicians, translators and administrative staff. The doctors and nurses are likely to be Danish and the rest of the staff, Greenlanders. The advantage of the health care system is the very close contact between the staff and the patients with many of the staff likely to be members of the local communities. Telemedicine is also an increasingly important facility in the health system particularly for the more remote health centres (9).

#### Health expenditure

Health care expenditure in Greenland totalled DKK 1066M in 2008 (or approximately US$ 197 million).^3^. On a per capita basis of expenditure, Greenland spent the lowest among the Nordic countries in 2008 at approximately 18,880 DKK or US$ 3,530 per person. [At the same time, Denmark spent 28,836DKK or US$ 5,391 on health per capita (8).] Overall, health care accounts for more than 18% of total government expenditure or 9.1% of Greenland's gross domestic product (8). A breakdown of the budget shows that about 47% is spent in the health districts, the national hospital in Nuuk accounts for 28%; 2% is dedicated to preventive efforts and surveillance and 12% is for treatments outside Greenland; 6% is used to transport patients (7).

### Alaska

#### Population

Out of a total population of close to 710,000 in Alaska, Inuit account for about 7% (50,000) or about half the number of American Indians and Alaskan natives living in Alaska (10). This percentage contrasts sharply with the rest of the United States where Native Americans comprise about 1% of the population. It is also important to note that the Alaskan Native population is dominant in the north and northwest of the state. The fact that many live in remote and isolated villages brings with it the challenges and constraints to health delivery familiar in other parts of the Inuit Arctic.

#### Geographic and political profile

Alaska is the largest state in the United States. However, with about half of Alaska's residents living in Anchorage, Alaska is also the least densely populated US state. About one-ninth of the state is owned by Alaskan natives as a result of the Alaska Native Claims Settlement Act which led to the creation of 12 regional and a number of local Native corporations. Much of the land mass is only accessible by air.

Inuit territory includes the North Slope Borough consisting of 7 villages served by the Arctic Slope Regional Corporation; Northwest Arctic Borough comprising 11 villages and Bering Straits Regional Corporation, which includes 16 villages. Barrow, Alaska in the North Slope Borough is the most northern US city.

#### Profile of health system

The federally funded Indian Health Service (IHS) is the principal funder of health care for Alaska Natives including Inuit.

#### Health challenges

Cancer, heart disease, accidents including drowning, suicide and substance abuse are the leading causes of death. Issues preventing Alaska Natives from receiving quality medical care include cultural barriers, geographic isolation, inadequate water systems and sewage disposal and low income. There is inadequate data on the health and well-being of Alaskan natives including Inuit and this has been recognized as a potential problem for the accurate measurement of health inequalities and the accurate application of programs and funds (2).

Inadequate data on the health and well-being of American Indians and Alaskan natives poses potential problems for the accurate measurement of health inequalities as well the accurate application of programs and funds towards disparity in health status and care … Unique patterns of disease or behavioral characteristics correlated with disease may not be identified or addressed. Insufficient and inaccurate data prohibit comparisons among tribes, underserved populations, and impair the ability to determine whether a health problem is emerging or simply previously undocumented. In terms of funding, agencies may not support selected programs because health conditions are unrecognized within collected data. Limited data from some American Indian and Alaskan Native populations may be generalized to other or even all American Indian and Alaskan Native communities. This results in the application of programs, resources, and funding to problems which may or may not exist in all communities. (2)

#### Responsible authorities for Alaska Native health services

The federal government, through the IHS, is required to provide health care services for Alaskan natives. From 1970 onwards, Alaska natives, including Inuit, developed health care organizations under self-determination legislation and assumed ownership of health services with regional and village corporations providing the services through compacts and contracts negotiated under the Indian Self-Determination and Education Assistance Act of 1975. Self-governance legislation in 1994 provided for perpetual compact agreements between the US Department of Health and Human Service and tribal programs. Since 1998, all Alaska Native health care is provided by Alaska Native organizations (11).

The Alaska Tribal Health System is a voluntary affiliation of nearly 40 Alaskan tribes and tribal organizations providing health services. The Alaska Tribal Health Compact is the umbrella agreement that sets out the terms and conditions of government-to-government relations through the IHS. Twenty-two tribes and tribal organizations belong to the compact which authorizes tribes and native health organizations to operate health and health-related programs. Organizations that belong to the Compact include organizations responsible for providing some of the health services to Inuit. These are the Arctic Slope Native Association, Maniilaq Association, Norton Sound Health Corporation, Seldovia Village Tribe and the Yukon-Kuskokwim Health Corporation. The Alaska Native Health Board (ANHB) is recognized as the state-wide advocacy organization on Alaska Native health issues.

Each tribal health organization retains its autonomy with regard to health priorities, services and policies. The entire system serves approximately 130,000 Alaska natives. The medical care services include small community primary care centres, sub-regional mid-level care centres, 6 regional hospitals including the Samuel Simmonds Memorial Hospital in the North Slope Borough, the Alaska Native Medical Center tertiary care and referrals to private medical providers and other states.

The Alaska Native Tribal Health Consortium, a not-for-profit tribal health organization managed by Alaska Native tribal governments and their regional health organizations, was created in 1997 to provide state-wide native health services and to support tribal health organizations and communities. It provides tertiary and specialty medical, community health and research, environmental health and engineering, health information technology services and professional recruitment.

Dental facilities are provided through 14 regional hub dental clinics as well as mobile dental services which visit villages.

#### Health expenditure

As in other parts of the Arctic, the geography and climate contribute to higher medical costs and abiding constraints in ensuring appropriate and adequate staff for health services. Health care expenditures in 2004 were among the highest in the United States at US$ 6,450 per capita compared to the national average of US$ 5,283 (12).

As the funding provided by IHS is inadequate, the native health care system also relies on Medicare, Medicaid and private insurance payers to supplement the annual budget (12).

### Canada

#### Population

According to Aboriginal and Northern Development Canada, there are 50,485 Inuit across the country. This is approximately 4% of the total Canadian population who identify themselves as an Aboriginal person. The majority of Inuit in Canada – about 78% – live in one of 4 regions within Inuit Nunangat (Inuit homeland) which is the region stretching from Labrador in the east to the Northwest Territories: Nunavut (24,635 Inuit according to the 2006 Census); Nunavik (9,565 Inuit); Inuvialuit in the Northwest Territories (3,115 Inuit) and Nunatsiavut (2,160 Inuit). Inuit are the majority of the population in all 4 regions accounting for 90% of the total population in Nunavik, 89% in Nunatsiavut, 84% in Nunavut and 55% in the Inuvialuit region (13).

#### Geographic and political profile

More than three-quarters of Canadian Inuit live in their traditional Arctic homeland. All traditional Inuit lands in Canada are covered by some sort of land claims agreement providing for regional autonomy. The land claims settlement for Quebec Inuit through the James Bay and Northern Quebec Agreement in 1975, which established the region of Nunavik, was the first. The Labrador Inuit submitted their land claim in 1977 although it was not until 2005 that the land settlement claim was signed leading to the establishment of Nunatsiavut. The Tunngavik Federation of Nunavut (now the Nunavut Tunngavik Inc.) was incorporated in 1982 with the Nunavut Final Agreement approved 10 years later and the Nunavut Land Claims Agreement signed in 1993. Nunavut, as a territorial entity was established in 1999. The western Canadian Inuit, the Inuvialuit, who are in the Northwest Territories are represented by the Inuvialuit Regional Corporation and received a comprehensive land claims settlement in 1984.

#### Health challenges

Various sources indicate that very little health information is collected on the Canadian Inuit population outside Non-Insured Health Benefit records although as noted below, even these records do not reveal much information. Some provinces and territories collect health statistics on the Aboriginal population but their methods, including specific health indicators, differ. The inadequacy of Inuit-specific data and systematic research on Inuit health has a significant impact on the ability of health providers and Inuit communities and organizations to monitor the Inuit health care system (2). Reporting on the health status of Canadian Inuit is difficult due to a lack of comprehensive and comparable information and data. This results in an incomplete picture of Inuit health status (14).

Key data issues for Inuit are: the lack of funding and infrastructure to conduct their own population-level survey research; confidentiality and validity in small sample sizes; lack of an Inuit identifier in most provincial and territorial administrative data; lack of Inuit specific rather than geographically-based socio-economic data and information on health determinants; difficulties accessing university-based research funding and finding support for community-driven applied research; and lack of data on urban Inuit. (2)

That said, what is known points to a lower health status among Inuit compared to other Canadians. In particular, Inuit are experiencing high rates of suicide at 11 times greater than the overall Canadian rate; the incidence of tuberculosis is up to 170 times higher than non-Aboriginals and twice the rate estimated in 2004; lung cancer death rates for Canadian Inuit are the highest in the world (15); mental health and substance abuse are major concerns.

#### Profile of health system

The national health system is universal coverage with health expenditure largely funded by the government although there is some limited private funding through insurance or out-of-pocket payments.

Responsibility for Inuit health services is divided between the federal and provincial/territory governments. The federal government's role includes setting and administering national principles for the system under the *Canada Health Act*; financial support to the provinces and territories; and several other functions, including the direct delivery of primary and supplementary services to certain groups of people; public health programs; health protection; and funding for health research and health information activities. The federal government provides cash and tax transfers to the provinces and territories in support of health through the Canada Health Transfer.

The provinces and territories administer and deliver most of Canada's health care services. The provincial and territorial governments fund these services with assistance from federal cash and tax transfers. The role of the provincial and territorial governments in health care includes administering their health insurance plans; planning, paying for and evaluating hospital care, physician care, allied health care, prescription drug care in hospitals and public health; and negotiating fee schedules for health professionals (16).

Most federal health care programs for Inuit are funded through First Nations and Inuit Health (FNIH) in Health Canada. From FNIH, Inuit receive Non-Insured Health Benefits (NIHB) (dental, drug, vision, crisis mental health services, medical equipment and supplies and medical transportation benefits not available through provincial or territorial systems); and targeted community-based public health and health promotion programs. The NIHB program, one of the biggest federal expenditures, is administered in some regions by or for Health Canada, which does not separate Inuit expenditures. It is therefore difficult to analyze the effectiveness of this funding for Inuit. Other federal departments provide funding to support Inuit health, such as Aboriginal Head Start and Childcare Initiatives (15).

#### Provincial and territorial delivery systems relevant to Canadian Inuit

In 1988, the Government of the Northwest Territories (GNWT) and in 1999, the Government of Nunavut (GN) made Transfer Agreements with the federal government, accepting responsibility for health care services to all residents, including most programs targeted towards Inuit and First Nations. Inuvialuit receive health care services through regional boards established by the GNWT. Inuit in Nunavut receive health care services through a centralized system that serves all residents. Inuit in Nunavik receive health care services through the Nunavik Regional Board of Health and Social Services. The Board, which is governed by Inuit, was established under the James Bay and Northern Québec Agreement. Health care funds, provincial and some federal, flow from the Québec government to the Board. Other federal funding goes directly to the Board through contribution agreements (15). The Nunatsiavut Government's Department of Health and Social Development is responsible for the promotion of health and social needs of the beneficiaries of the Labrador Inuit land claim. The delivery model is complex with Primary Care and the Management of Communicable Disease Control, the responsibility of Labrador Grenfell Health, one of 4 provincial health authorities.

In the case of all the Inuit territories, travel outside an individual's community or region is required for many health care services and treatments. With none of the northern Inuit communities having year-round road access, patients need to be flown out for medical consultations or treatment. Weather conditions can delay departures (17).

#### Health expenditure

Total health expenditure in Canada in 2009 was CAD$ 182.1 billion and estimated expenditures in 2010 and 2011 to be CAD$ 192.9 billion and $ 200.5 billion, respectively. Health expenditure per capita was CAD$ 5,401 in 2009 with forecasts of CAD$ 5,654 and CAD$ 5,811 for 2010 and 2011, respectively (18).

Within the publicly funded health care system, health expenditures vary across the provinces and territories. This is, in part, due to demographics, including population density and location as well as geography. Health expenditure per capita is highest in the territories because of their large geographical areas and low population densities. In 2011, for the territories, the health expenditure-to-territorial GDP ratio is 21.3% for Nunavut, 12.8% for the Yukon and 8.8% for the Northwest Territories (18).

### Chukotka (Russia)

#### Population

There are only about 1,750 Inuit among Russia's indigenous peoples who represent just over 19% of Russia's 143 million people.

#### Geographic and political profile

In the post-World War II period, intensive migration from central Russia into Chuktoka had a significant impact on the situation for Chukotkan Inuit. They increasingly became a minority as the population almost doubled as a result of the influx. However, with the breakup of the USSR in the 1990s, there was a reversal of the inward flow as people from other parts of former USSR returned to their places of origin to become citizens of the newly independent states. As a result, the population of Chukotka fell from 164,000 in 1989 to 74,000 at the time of the 2002 Census (4).

#### Health challenges

Mortality rates from accidents, homicide and suicide are very high in the northern parts of Russia and especially among the indigenous population. Violent deaths and alcohol abuse are the main causes of the shortened life expectancy in the north (indigenous and non-indigenous). Life expectancy for the numerically small indigenous peoples of the north was estimated in 2004 at 45 years for males and 55 years for females compared to the all-Russia rate of 61 years for males and 74 years for females (4). It should be noted however that the health status of the Russian population in general declined after the establishment of the Russian Federation in 1991. Rates of tuberculosis [prevalence at 69 per 100,000 population (4)], cancer and heart disease are the highest of all industrialized countries. It also has a significant HIV/AIDS epidemic with a prevalence rate of 11 infections for every 1,000 adults. However, there has been a significant growth in public health spending over the past decade and a large-scale health care reform program was announced in early 2011.

#### Profile of health system

The Constitution of the Russian Federation guarantees free medical care for every Russian citizen. However, the funding of health care is through various formal and informal systems: tax-based medical insurance; employer contributions to the Medical Insurance Fund; voluntary health insurance payments; out-of-pocket expenses; and “under the counter” payments to doctors and institutions.

#### Responsible authorities for health services

The health care system in the Russian Federation is a decentralized administrative structure divided into federal, regional (oblast-level) and municipal (rayon-level) administrative levels. One third of the population receives primary care through work-related clinics and hospitals. As regional budgets fund the bulk of health care costs, there is wide disparity between standards and health indicators across the country.

The delivery of health services in Russia is a federal, regional and municipal responsibility, carried out in accordance with federal and regional regulations and funded through multiple sources (for example, the federal budget and transfers, regional budgets, and health insurance). However, with the majority of taxes used to fund the health service paid direct to the regions, the poorer regions have less for health care. As a result, the quality of and accessibility to health services is influenced by where you live. The reform of regional health systems is a major challenge for the country.

The health care facilities vary but the principle ones are rural health posts which offer basic health checks and facilities serving smaller communities; health centres which serve larger rural populations and offer primary care services as well as minor surgery; urban polyclinics which provide a range of general and specialist services; and special focus polyclinics for children.

#### Health expenditure

While the current status of Russia's National Health Accounts was not available, 2008 figures show that the total expenditure on health per capita was US$ 866. Total expenditure on health in 2008 as a percentage of GDP was 5.2% (19).

### Comparing the systems

Table III provides a snapshot of the differences between each of the 4 Inuit countries in terms of the variations between health systems in place and health expenditure per capita on an annual basis. In the fourth column, the most recent life expectancy indicator is listed. As can be seen, it is not possible to draw linkages between the different characteristics of the respective health systems, the corresponding financial investment and the system's effectiveness in adequately serving Inuit health needs. There are a number of reasons for this inconclusiveness. First, as noted in several earlier sections, the collection of Inuit-specific health data in Alaska, Russia and Canada, is less than adequate in a number of ways making it difficult to identify those issues which are particularly relevant to drivers and determinants affecting Inuit health. Second, the data that are available do not necessarily provide a feasible point of comparison in terms of methodology and timing of the data collection.

**Table III:** Health system comparisons and outcomes

Country	Health system	Health expenditure per capita per annum (USD)	Life expectancy
Greenland	Universal healthcare	$ 3,530 (2008)	68 years (2008/WHO)
Alaska	Federal Indian Health Service complemented by public and private insurance	$ 6,450 (2004)*	69.4 years (Alaskan Natives/2000 Census)
		National $4,671	
Canada**	Universal healthcare complemented by limited private funding	Northwest Territories $ 8,923 (CAD – 2005–2009)	Inuvialuit: 70.3 years**
		Nunavut $ 11,801 (CAD – 2005–2009)	Nunavut: 68.2 years***
		National $ 4,915 (CAD – 2005–2009)	All Inuit regions: 66.9 years**
			National: 79.5 years***
Russia	Free medical care funded through insurance, OOP and personal payments	National $ 866 (2008)	National: 61 years (2008)
			Inuit: 58.6 (00–04)

* This is state-wide expenditure and not divided into the per capita amount spent by HIS.

** The 2005–2009 data have been used instead of the more recent data published by the Canadian Institute of Health Information in 2011 and referenced elsewhere in this document. The preference for the 2005–2009 figures in this table is to provide a closer comparison with the latest figures available for the other 3 countries.

*** Life expectancy for Inuit and non-Inuit living in Canadian Inuit communities, 2001; ITK Inuit Statistical Profile, 2008; http://www.itk.ca/sites/default/files/InuitStatisticalProfile2008_0.pdf.

That said, the contrasting variations between health expenditure per capita and life expectancy outcomes warrant more detailed investigation to determine why the funds invested are not resulting in comparably similar health outcomes. This is particularly noticeable in Nunavut, Canada where the expenditure is the highest across all Inuit regions yet the life expectancy outcome is less than Greenland where the least amount (with the exception of Russia) is spent on an annual basis. There will be a number of factors influencing this variation not least being the location of population and health centres, the costs involved in transporting patients to southern health centres, the isolation of communities, prevailing economic conditions and opportunities for economic activity, all of which affect the effectiveness of the formal health system.

Overall, despite the variations in the degree of accessibility, universality and expenditure, none of the health systems is adequately meeting the health challenges if health indicators are benchmarks for judging success. However, it is important to remember when assessing the quality or otherwise of individual health systems that physical and mental health is not simply the product of good, inadequate or underfunded health systems. Good health and well-being for Inuit across the Arctic is driven by a number of factors including social determinants such as cultural vibrancy, application of traditional knowledge, employment and education opportunities. Physical and geographical constraints such as isolation and distance to health and education facilities also play a major role in affecting Inuit health as do other factors such as housing, environmental contamination, unsafe drinking water, inadequate sewerage and the effects of climate change.

This has been recognized by the Inuit Circumpolar Council, which has developed a 4-year Circumpolar Inuit Health Strategy (2010–2014) with the objective of improving Inuit health and wellness across the Arctic through its advocacy and research. The organization recognizes that approaching Inuit health and wellness requires a full appreciation of the various, and in some cases, unique factors impacting on Inuit well-being.

The priority issues for ICC are the centrality of Inuit culture and traditional knowledge, access to traditional or country foods and food security, the impact of climate change and other environmental factors, addressing substance abuse and improving access to health facilities.

Traditional knowledge and medicine is a key component in underpinning Inuit health and wellness. Alienation from and changes to Inuit culture have been cited as major contributors to both physical and mental health problems. The impact of climate change on the Arctic is well recognized and well documented and the effect on Inuit health and wellness in particular is expected to be felt in a number of areas ranging from increased injuries due to fragile and changing ice behavior through to changes in animal and bird migration patterns and increased exposure to environmental contaminants. Other longstanding environmental issues include housing, water supply and sanitation. Inadequate, overcrowded and damp housing often polluted with tobacco smoke is contributing to high rates of respiratory illness and violence.

Factors that contribute to food insecurity include the high cost of food in remote communities, the cost of hunting, a limited amount of income and inadequate government support. An additional factor is the concentration of contaminants in animals, which comprise much of the traditional diet for Inuit.

Alcoholism has been and continues to be one of the most acute problems for many Inuit communities. Identified as a primary health and social concern in Inuit communities because of its devastating consequences, alcohol and other substance abuse is both a contributor to behavioral and mental problems and also a symptom of existing trauma – both at the individual and community levels.

Access to appropriate health facilities and support services is a longstanding challenge stemming in part from the inherent difficulties of providing services and attracting staff to what are often remote and harsh Arctic locations. The challenge has 2 parts: getting the diagnosis right and then making sure that the right treatment is provided for the right length of time.

The cost of delivering health care across the Arctic is significant although the outcomes often do not reflect the significant size of the investment, again because of the overarching cost of doing any business in the Arctic. The remoteness also affects the availability of appropriately trained health workers who are also prepared to stay. As seen in Greenland, one of the positives of the Greenlandic health care system is the fact that many of the people working in the system are also a part of the local community and so understand who they are working with.

While health is much more than statistics and indicators, where there is inadequate Inuit-specific data, determining existing and future health system needs and priorities for investment will be constrained and distorted by this absence of data.

## Conclusions

Despite the variations in the health systems as well as national political and economic approaches, none is adequately addressing Inuit health needs. All Inuit populations still have significant gaps between their health status and those of broader national populations. Health expenditure, with the exception of Russia, is high compared to the national average but this does not correlate with improved health outcomes.

Overall, however, meaningful measurement and evaluation of the effectiveness of the respective health systems is severely hindered by the lack of relevant, Inuit-specific health data. The inadequacy and in a number of cases absence of relevant data hinders the design and development of a better and arguably more effective approach to delivering health services to Inuit, particularly in Chukotka, Canada and Alaska, where the inadequacy of Inuit-specific data was widespread.

## Footnotes

1 For the purposes of this article, the term “Inuit” will be used when referring to the different Inuit populations in Canada, Greenland, Alaska (USA) and Chukotka (Russia) although locally they may be described as Inuit (Canada), Kalallit (Greenland), Eskimo (Alaska and Chukotka), Iñupiat (Alaska), and Yupik (Alaska and Chukotka).

2 WHO does not include Greenland within its online list of country health statistics and there is no reference to Greenland within WHO's statistics on Denmark.

3 Health care expenditure comprises both private and public expenditure which reflects the OECD's system of health accounts.
